# Reproductive Toxicity of 3,4-dichloroaniline (3,4-DCA) on Javanese Medaka (*Oryzias* *javanicus*, Bleeker 1854)

**DOI:** 10.3390/ani11030798

**Published:** 2021-03-12

**Authors:** Musa Adamu Ibrahim, Syaizwan Zahmir Zulkifli, Mohammad Noor Amal Azmai, Ferdaus Mohamat-Yusuff, Ahmad Ismail

**Affiliations:** 1 Department of Biology, Faculty of Science, Universiti Putra Malaysia, UPM Serdang, Selangor 43400, Malaysia; moosaad8@gmail.com (M.A.I.); mnamal@upm.edu.my (M.N.A.A.); aismail@upm.edu.my (A.I.); 2 Department of Biological Sciences, Faculty of Science, University of Maiduguri, Maiduguri 600230, Nigeria; 3 International Institute of Aquaculture and Aquatic Sciences (i-AQUAS), Universiti Putra Malaysia, Batu 7, Jalan Kemang 6, Teluk Kemang, Port Dickson 71050, Malaysia; ferdius@upm.edu.my; 4 Institute of Bioscience, Universiti Putra Malaysia, UPM Serdang, Selangor 43400, Malaysia; 5 Department of Environmental Sciences, Faculty of Environmental Studies, Universiti Putra Malaysia, UPM Serdang, Selangor 43400, Malaysia

**Keywords:** reproduction, fecundity, gonad, histopathology, fertilisation

## Abstract

**Simple Summary:**

Compound 3,4-dichloroaniline (3,4-DCA) is a degradation product of some herbicide and intermediate industrial chemicals of many products. Unregulated use of 3,4-DCA affects the reproduction of non-target organisms. In this study, the potential reproductive toxicity of 3,4-DCA on Javanese medaka, a native sentinel organism distributed around aquatic ecosystems of Southern Asia, was evaluated. Low fecundity was observed in female exposed fish to 250 µg/L of 3,4-DCA. Gonad tissue alternation and interference of gametes development were observed both in male and female exposed fish. The response pattern indicated that a low concentration of 3,4-DCA exerted endocrine disruption in Javanese medaka. This finding would provide additional data on the toxicity effect of 3,4-DCA, and it will strengthen the potential of native species in monitoring local and regional aquatic environments.

**Abstract:**

Compound 3,4-dichloroaniline (3,4-DCA) is a metabolite of several urea herbicides and intermediate chemical of several industrial products. Moreover, 3,4-DCA has been frequently detected in aquatic ecosystems around the world. This aniline is more toxic than the parent chemicals, and it affects non-target organisms. This study evaluated a 21-day reproductive response of an emerging aquatic vertebrate model, Javanese medaka (*Oryzias javanicus*), exposed to 3,4-DCA. Fecundity and gonads histopathology were observed. The spawning rate and fertilisation reduced significantly in the highest exposed-group (250 µg/L). Gonadosomatic index (GSI) was significantly low in females exposed to 250 µg/L. No substantial structural alteration of male gonads. However, oocyte development and ovarian cell structure were disrupted in 250 µg/L exposed females. The gonadal developmental was not affected in the males; however, a significant reduction in the developmental of female gonads was observed at 250 µg/L. These results show that 3,4-DCA interfere with the reproduction of Javanese medaka through fecundity and alteration of gonadal tissues.

## 1. Introduction

The aniline, 3,4-dichloroaniline (3,4-DCA), is a metabolite of diuron and some herbicides [1] that reach the aquatic ecosystem in considerable concentrations through agriculture and industrial production of other parent chemicals [2,3]. In water, the concentration of 3,4-DCA is usually more than diuron in aquatic organisms; fish and crustaceans are susceptible to 3,4-DCA [4,5]. The Organisation for Economic Co-operation and Development (OECD) recognised 3,4-DCA as a reference chemical for toxicity testing [1,6].

Anilines prevent the metabolic pathway of unspecific membrane in the cell [1,6]. Embryotoxicity was observed in Javanese medaka [7] and zebrafish (*Danio rerio*) exposed to sublethal concentration of 3,4-DCA [8] embryos exposed to 3,4-DCA. Moreover, 3,4-DCA has prolonged toxicity in different organisms [9,10]. It is also toxic to humans [9]. Various studies have demonstrated that 3,4-DCA has nephrotoxicity and reproductive toxicity to mammals [2]. In addition, 3,4-DCA, as a metabolite, is frequently detected in the environmental compartments compared to its mother chemicals (diuron, propanil, etc.) [2]. Concentrations of 567 μg/L in water and 119 mg/kg in soil were detected in some environmental samples [11]. Aquatic pollutants (endocrine-disrupting chemicals; EDCs) can affect reproduction, development, immunity, and other physiological mechanisms [12,13], which eventually threaten fish’s survival. Reproduction and development in fish are essential factors that predict its population dynamics [14].

Numerous anilines can disrupt reproductive and endocrine processes [3]. Importantly, 3,4-DCA acts as an antagonist; it competes with testosterone to bind with the androgen receptor (AR) [15]. At 100 and 200–400 μg/L, 3,4-DCA affected the secondary sex characters (male colour intensity and courtship behaviour) and androgen of *Gasterosteus aculeatus* 96-h post-exposure [1]. It was reported that 3,4-DCA is an endocrine-disrupting compound (EDCs) [3,7,8,16].

There is a worldwide increase in interest in EDCs. The quantity and types of EDCs released into the aquatic environment from different sources, such as agricultural runoff, waste treatment plants, and paper mill plants, can affect the reproduction of aquatic organisms [17]. EDCs affect the development and activities of both male and female reproductive structures [18]. However, the oestrogenic activities and health status of organisms exposed to some EDCs are transgenerational [12,16,19]. Estrogenic EDCs originate from both anthropogenic and natural sources [12].

Javanese medaka (*Oryzias javanicus*) inhabit estuarine ecosystems of Peninsular Malaysia, Singapore, Indonesia, and Thailand [7,20,21]. Javanese medaka possessed significant features that make it relevant to the widely used model fish [21,22,23,24]. To date, several studies on the toxicity of various chemicals on Javanese medaka have been conducted [25]. Generally, there is limited research on the reproduction of Javanese medaka. The size of Javanese medaka, concentration, and exposure period (usually ≤21 days) are adequate for toxicity response in the fish short-term reproduction assay (FSTRA) of endocrine-active substances. Imai et al. [26] observed that at least 16 ng/L of one of the well-known endocrine active chemicals, 7β-estradiol, disrupt the reproduction of Javanese medaka (*Oryzias javanicus*).

However, as an emerging model fish in Southern Asia, there are limited toxicological studies of organic chemicals on its reproduction. There is a limited report on the effects of 3,4-DCA on estuarine species [27] as compared to freshwater [19,28,29,30] and marine organisms. The differences in sensitivity of organisms to toxicant would provide possible response across diverse ecosystems. Therefore, more toxicological data is needed to strengthen the response pattern of euryhaline species to 3,4-DCA. Thus, this study aimed to evaluate the fecundity and alterations of gonads changes of Javanese medaka exposed to sublethal concentration of 3,4-DCA.

## 2. Materials and Methods

### 2.1. Source of Chemicals

Analar grade of 3,4-dichloroaniline (CAS: 95-76-1, purity: 98%) was purchased from Sigma-Aldrich (St. Louis, MO, USA). The solvent (DMSO, CAS: 67-68-5, purity: 99.9%) was obtained from Friedemann Schmidt Chemical (Kajang, Selangor, Malaysia). 

### 2.2. Javanese Medaka Maintenance

F_2_-generation (laboratory culture) of wild strains of Javanese medaka were collected from a water recirculation system in Javanese Medaka Mass Culture Laboratory, Department of Biology, Faculty of Science, Universiti Putra Malaysia. Water condition was maintained at 26 ± 1 °C, pH: 7.8–8.0, DO: 5.5–6.0 mg/L, salinity: 0.54 ± 0.05‰, light cycle: 14 h light:10 h dark. The fish were fed twice daily (9:30–10:00 a.m. and 5:00–5:30 p.m.) ad libitum with freshly hatched nauplii of *Artemia* sp. The water recirculation system was cleaned: ~80% of the water was changed at intervals of 3 weeks to avoid unnecessary removal of beneficial microflora that prevent accumulation of toxic ammonia and nitrite from excreta and uneaten food debris.

### 2.3. Selection of Reproductively Active Adults

Reproductively active adults (3 months post-hatched, total length: 31.5 ± 0.2 mm; body weight: 0.26 ± 0.02 g) were selected in a ratio of 1 male:3 female (12 fish) per glass tank in all the treatment groups (i.e., a total of 252 fish). 

### 2.4. Preparation of Test Chemicals and Exposure

Five exposure groups (10, 25, 50, 125, and 250 µg/L) of 3,4-DCA were prepared from a stock concentration (100 mg/L). The experiment was conducted along with solvent control (0.01% *v/v* DMSO) and dilution water control (dechlorinated tap water) [31]. The highest concentration was selected based on the no observed effect concentration (NOEC) of the 96 h LC_50_ acute toxicity. The treatments (5 exposure groups and 2 control groups) were done in triplicates with glass aquaria (37.5 cm × 17.5 cm × 18 cm) containing 6.0 L of a given treatment. The treatments were changed every 48 h. The exposure duration was 21 days.

### 2.5. Morphometric and Somatic Indices

The fish were euthanised by immersion in 75 mg/L of tricaine methanesulfonate (MS-222) for ~1–2 min and blotted with paper towels on the day of termination (21 days post-exposure). The average total body length (mm), body weight (mg), the weight of gonads, and liver weight of the fish in all the treatment groups were determined. Hepatosomatic index (HSI = [liver weight/body weight] × 100), gonadosomatic index (GSI = [gonad weight/body weight] × 100), and the condition factor ((K) = [W100/L^3^]; where W = body weight (mg) and L = total length (mm)) (Bannister, 1976) were determined. The gonads were removed and weighed with an analytical balance (±0.0001 g).

### 2.6. Fecundity

Eggs attached to the urogenital opening and the bottom of the glass tanks were collected with a plastic pipette ~30–45 min after the light turned on. The total number of spawned eggs, fertilisation (mean fertilised eggs), and fertility rate (= [fertilised eggs/total eggs spawned] × 100) of each treatment tank was determined daily. 

### 2.7. Gonad Histopathology

Whole body of individual of the euthanised fish (one male and one female/replicate from each experimental unit: glass tank) were immersed in Davidson’s solution for 24 h, later transferred to 10% buffered formalin solution. The fish were dehydrated and embedded individually due to their sizes with Technovit 7100 (Heraeus Kuizer GmbH, Wehrheim, Germany). The transverse section (⁓5 μm) of the tissues was prepared with a microtome. Sectioned-tissues were stained with haematoxylin and eosin (H & E) and mounted with Eukitt (O. Kinder, Freiburg, Germany). The sections were examined under a light microscope.

### 2.8. Statistical Analysis

The normality was verified using Shapiro–Wilk normality test. One-way ANOVA followed by Dunnett’s multiple comparisons test was performed between controls and the treatment groups, where the assumption failed, Kruskal–Wallis test was used. All the analyses were performed using GraphPad Prism version 7.00 for Windows (GraphPad Software, La Jolla, CA, USA) at 95% confidence interval (*p* < 0.05). Values are presented as mean ± standard error of mean ($\bar{x}$ ± SEM).

## 3. Results

**Figure 1:**
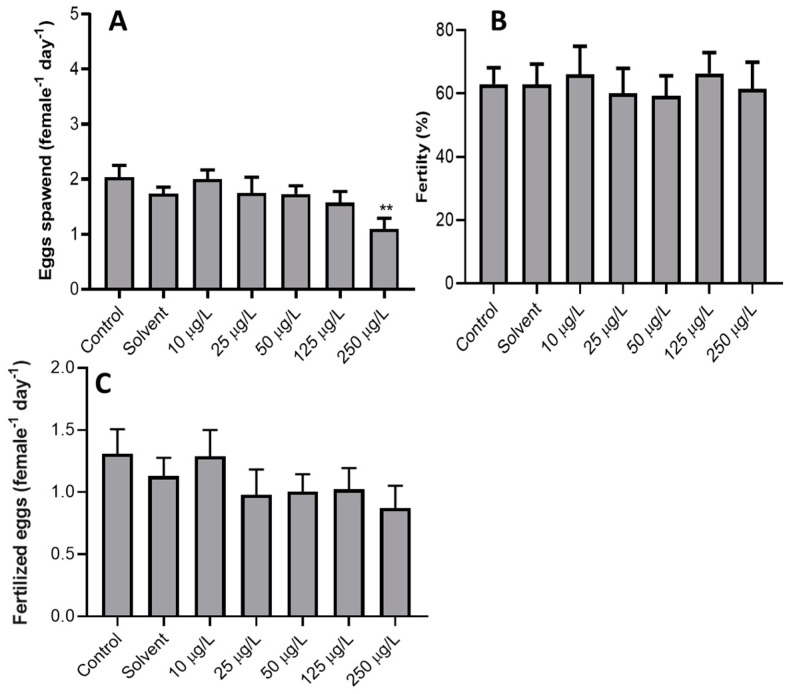
Fecundity of Javanese medaka (n = 3) exposed to sublethal concentration of 3,4-dichloroaniline (3,4-DCA) at 21 days exposure: (**A**) total eggs spawned, (**B**) fertility, and (**C**) fertilised eggs ** indicates a significant difference with control at *p* < 0.01. Error bar = ± standard error of mean (SEM).

Figure 1A shows that the average total eggs spawned significantly reduced (*p* = 0.007) at 250 µg/L compared with the control group. No significant difference in the fertility (%) (*p* = 0.990) (Figure 1B) and number of fertilised eggs (*p* = 0.557) between all the treatment groups (Figure 1C) was observed.

There was no significant difference in the average total body length (*p* = 0.596) and body weight (*p* = 0.505) between the treatment groups. However, the condition factor (K) did not substantially differ in male and female fish from different treatment groups (Table 1). Reduction in K implies depletion in reserved energy in fish due to stress factor, thereby disrupting normal physiological processes.

Table 2 shows that there was no significant difference in the HSI of males (*p* = 0.995) and females (*p* = 0.054) from all the treatment groups. The GSI of males in the exposed and control groups were not significantly different (*p* = 0.068). However, the GSI of females exposed to 250 µg/L was significantly low (*p* = 0.012) between all the treatment groups.

**Figure 2:**
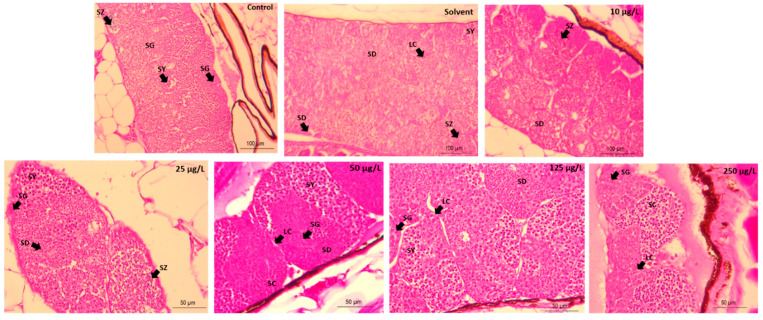
Microphotograph (H&E stain, mag. ×40) of Javanese medaka testes at 21 days post-exposure to sublethal concentration of 3,4-DCA. SC, Sertoli cell; LC, Leydig cell; IPF, intravascular proteinaceous fluid, spermatogonia; SG, spermatocytes; SY, spermatids; SD, spermatozoa; SZ.

The gonads show all developmental stages of spermatogenic cells (spermatogonia; SG, spermatocytes; SY, spermatids; SD, spermatozoa; SZ) in all the treatment groups. However, there was a proliferation of Leydig cells in the 125 µg/L exposed-group. There were substantial SG and SC domination at the highest exposure level (250 µg/L) (Figure 2).

**Figure 3:**
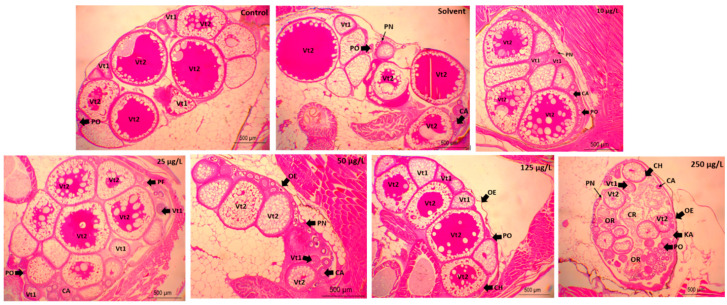
Microphotograph (H&E stained, ×40) of Javanese medaka ovaries at 21 days post-exposure to sublethal concentration of 3,4-DCA. AO, atretic oocyte OE, ovarian epithelium; BV, blood vessels; PF, primordial follicle; LF, large follicle; GR, granulosa cell; OC, ovarian cavity; OE, ovarian epithelium; OC, ovarian cavity; GF, growing follicle; LF, large follicle; CH, chorion; DM, Vt1, primary vitellogenic oocyte; Vt2, secondary vitellogenic oocyte, PN, perinucleolar oocytes; CA, cortical alveolar oocytes; PF, postovulatory follicle.

The development stages of the oocytes from different treatment groups show synchronous development in control groups (dilution water and solvent) and 25 and 125 µg/L, while 50 and 250 µg/L oocytes indicate synchronous development. However, at 250 µg/L, the secondary vitellogenic stage (Vt2) dominates the ovary, no primary vitellogenic stage (Vt1) was observed, i.e., degeneration of ovarian cells and high proportion of perinucleolar oocytes (Figure 3).

Table 3 shows the gonadal development stage (median) of both sexes and ovarian tissue lesions from different treatment groups. The median stage was low (late spermatogenic; stage 3) at 50 and 250 µg/L. However, there was no significant difference (*p* = 0.301) between all the treatment groups. However, development in females exposed to 250 µg/L were significantly low (*p* = 0.069) (mid-development; stage 2). The proportion (%) of atretic oocytes was significantly higher (*p* = 0.034) at 250 µg/L, however, there was no significant difference in oocyte retraction (*p* = 0.141) and karyoptic clumping (*p* = 0.222) in all the treatment groups.

**Figure 4:**
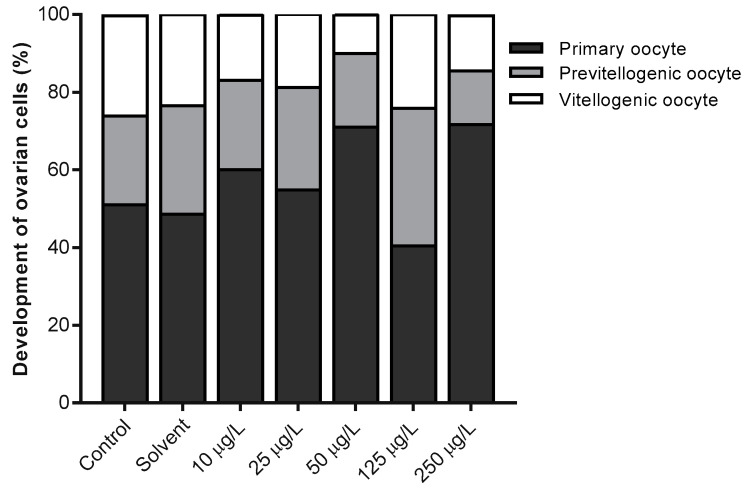
Development of oocyte in Javanese medaka (n = 3) at 21 days post-exposure to 3,4-DCA.

Figure 4 shows the proportion (%) of the major developmental stages of the female fish’s’ ovarian cells from different groups. The result shows that there was no significant difference in the number of primary oocytes (*p* = 0.244), previtellogenic oocytes (*p* = 0.367), and vitellogenic oocytes (*p* = 0.174) in all the treatment groups. The highest number of primary oocytes was 71.67 ± 13.3% in 250 µg/L exposed group. For the previtellogenic oocyte, 125 µg/L was the highest (35.37 ± 5.2%), and the lowest vitellogenic oocyte was 10 ± 2.7% at 50 µg/L.

## 4. Discussion

In this study, the reproductive response of Javanese medaka exposed to sublethal concentration of 3,4-DCA was evaluated. Reproduction is a vital process that regulates population dynamics and functioning ecological communities. Biomarkers like GSI, fecundity rate, fertilisation rate, and eggs hatchability are used as diagnostics tools for the potential hostile effects of xenobiotics on fish reproduction [32,33,34]. At the same time, HSI indicates the health status of fish in a given environment. Estrogenic substances disrupt gonad morphology, fertility, fecundity, and behaviour [35]. The temporal (3 weeks) decrease in fecundity in Javanese medaka post-exposed to 3,4-DCA with both linear and non-linear patterns indicates toxicity stress that affects the normal physiological activities and a potential threat to its population structure. In this study, the fertility (%) of spawned eggs of Javanese medaka exposed to different treatments of 3,4-DCA was not significantly different. Fecundity is one of the factors that predict the functions of the gonads and mating behaviour in fish [36]. There was a decreased in reproduction of three euryhaline species, i.e., western mosquitofish (*Gambusia affinis*), sailfin molly (*Poecillia latipinna*), and least killifish (*Heterandria formosa*), in low salinity [37,38]. The result of this study is contrary to Glinka et al. [38], who reported that 5α dihydrotestosterone (an EDC) did not affect egg production of an estuarine fish, Mummichog (*Fundulus heteroclitus*) at low salinity (1.0–2.5‰). Homoeostasis to balance osmotic regulation and reproduction and other physiological activities might have been disrupted [37,38].

Similarly, in a 3-week exposure, 1.27–89.4 mg/L of 4-nonylphenol (NP) did not affect egg spawning and fertility of Japanese medaka [39]. In another study, 500 µg/L of 3-(4-Methylbenzylidene) camphor (4-MBC) causes reproductive toxicity by reducing fecundity and fertility of Japanese medaka [14]. In water flea (*Daphnia magna*), 3,4-DCA exerted reproductive toxicity at a concentration of 0.02 mg/L and 20 pg/L [40]. Increased in time of exposure influences the effect of low concentrations of endocrine-disrupting chemicals [41]. Additionally, 3,4-DCA interferes with hormonal activities and ovulation in zebrafish (*Danio rerio*) at 3-week post-exposure [28]. Similarly, in a viviparous fish, *Chapalichthys pardalis* exposed to 3,4-DCA at 3.3 and 2.5 mg/L lost all their embryos [42]. The osmoregulatory mechanism of homeostasis in Javanese medaka influenced the toxicity response to 3,4-DCA. 

The morphometric parameters (total body weight and total length) and K of Javanese medaka exposed to the sub-lethal concentrations of 3,4-DCA were not significantly affected in the tests in this study. This finding implied that the treatments tested had no significant effect on the morphometrics observed. In another study, octocrylene did not significantly affect body length and the total body weight of Japanese medaka at a concentration up to 500 µg/L [14]. Likewise, Imai et al. [43] reported that estrone (E1) had no effect on Javanese medaka on total body weight and length. The size of Javanese medaka, concentration, and exposure period (usually ≤21 days) are adequate for toxicity response in the fish short-term reproduction assay (FSTRA) of endocrine-active substances [44], as observed in this study and related studies reported in the literature. Chronic or full-lifecycle exposure to 3.4-DCA may exert responses from what is observed in this study.

*Oryzias javanicus* and *Oryzias dancena* revealed high adaptability to sudden changes of the ambient osmotic pressure in both directions, i.e., from freshwater to seawater or vice versa [45]. The “chloride cells” of the eel gills is typical of the euryhaline teleosts, and is the primary mechanisms involved in the active regulation of osmotic concentration [46]. The salinity of the exposure media affects the uptake and reproduction toxicity of EDCs on the exposed fish [38].

The exposure concentrations used in this study did not affect the HSI in both males and females as earlier reported in Javanese medaka exposed to concentrations up to 5000 ng/L estrone, an EDC [43]. The GSI of males and females exposed to the highest concentration (250 µg/L) was lower, which indicated a stress symptom. The decrease in GSI might be associated with endocrine-active substances [47,48]. Similarly, GSI was not affected in Javanese medaka exposed to estrone, an EDC [43]. The condition factor (K), HIS, and GSI provide information on anthropogenic stress and health [49,50]. However, the male GSI in this study is not in line with the finding of Pereira et al. [19] that observed a decrease in GSI of male Nile tilapia (*Oreochromis niloticus*) exposed to 200 ng/L 3,4-DCA for 25 days. The reduction in GSI, germ cell production, and increased gonadal tissue abrasion was detected in teleost fish after exposure to different metabolites of diuron [19].

Histological changes in the gonads of male and female exposed Javanese medaka reveal stress symptoms. Domination of some gametophytic cells in exposed males is attributed to 3,4-DCA toxicity stress during gametogenesis. Gametogenesis, spermiation, and spawning are essential indicators of reproduction status in living organisms [51]. Inhibition of the gametogenic cells is associated with endocrine-disrupting effects [48,52]. Histopathological damages in the liver and testis were also observed in animal models; mice [2], and rats 30 days after exposure to 3,4-DCA [2,15]. 

There was no considerable difference in the stages of gamete development in male Javanese medaka. Similarly, there was no difference in gametes’ development in male Nile tilapia (*Oreochromis niloticus*) collected from lakes contaminated by oestrogenic compounds [53]. Moreover, 3,4-DCA may exert toxicity of male gonadal cells [15]. In this study, the significant reduction in oocyte development was due to toxicity stress by 3,4-DCA during follicular growth and differentiation. However, gonad tissue abrasions in Mysid (*Mesopodopsis slabberi*) 48 h post-exposure to 3,4-DCA were observed [27]. The substantial dose-dependent increase in primary oocytes in this study is in line with Rodgers-Gray et al. [54], who reported that the gonads of wild roach (*Rutilus rutilus*) that were exposed to treated sewage effluent for 150 days were affected similarly.

The three different oogenic stages observed (primary, previtellogenic, and vitellogenic oocytes) were not affected in a concentration-dependent trend. Non-linear (non-monotonic) patterns of the oocyte development are associated with endocrine disruption [55] by 3,4-DCA during oogenesis, which interferes with homeostasis and hormonal activities. It was previously reported that the disintegration of vitellogenic oocytes in female gonads exposed to EDCs [41,56]. High proliferation of atretic oocytes at higher concentration signifies cytotoxicity effect of 3,4-DCA in the gonads. Increase in atresia in the gonads of zebrafish exposed to 4 ng/L of 17α-ethinylestradiol (EE2), 50 µg/L of Fadrozole, and their mixture was observed at 90 days post-exposure [35]. Histological alteration indicates the bioaccumulation of 3,4-DCA on the gonadal tissues [27]. In fish exposed to xenobiotics, atretic oocyte indicates a pathological disorder [35].

## 5. Conclusions

The endpoints reported in this study have demonstrated that the subchronic exposure of Javanese medaka to 3,4-dichloroaniline (3,4-DCA) affects fecundity and fertilisation and alters the structure and development of both male and female gonads in a non-monotonic (biphasic)-like pattern. The non-monotonic dose-response of some of the endpoints is associated with endocrine disruption observed in other aquatic organisms in similar studies. This effect might be reversible/irreversible or multigenerational, depending on the mode of action at the enzymatic pathways that need further studies.

## Figures and Tables

**Table 1:** Morphometric and condition factor of Javanese medaka (n = 3) at 21 days post-exposure to sublethal concentration of 3,4-dichloroaniline (3,4-DCA).

Concentration (µg/L)	Total Length (mm)	Body Weight (mg)	Condition Factor (K)
Control	31± 3.7	213.6 ± 11.9	0.72
Solvent	28± 0.4	214.6 ± 18.8	1.01
10	28± 1.2	209.8 ± 29.9	0.94
25	27 ± 0.6	209.1 ± 11.2	1.04
50	25 ± 0.3	233.2 ± 9.6	1.49
125	27 ± 0.3	210.3 ± 3.5	1.07
250	29 ± 1.5	260.3 ± 33.3	1.07

**Table 2:** Hepatosomatic (HSI) and gonadosomatic (GSI) of Javanese medaka (n = 3) at 21 days post-exposure to sublethal concentration of 3,4-DCA.

Concentration (µg/L)	HSI (%)		GSI (%)	
	Male	Female	Male	Female
Control	3.65 ± 0.9	4.14 ± 0.8	2.44 ± 0.1	4.19 ± 0.2
Solvent	3.89 ± 1.3	6.34 ± 0.4	3.55 ± 0.8	4.92 ± 0.4
10	4.12 ± 0.8	4.15 ± 0.8	2.78 ± 0.5	3.64± 0.5
25	4.39 ± 1.3	6.03 ± 0.4	3.94 ± 0.3	3.79 ± 0.6
50	4.62 ± 1.4	5.11 ± 0.4	2.87 ± 0.2	4.36 ± 0.4
125	3.87 ± 1.6	5.16 ± 0.7	2.12 ± 0.6	2.84 ± 0.4
250	4.55 ± 0.3	5.90 ± 0.5	1.99 ± 0.4	2.47 ± 0.3 *

* indicates a significant difference with both control at *p* < 0.05.

**Table 3:** Gonadal staging and ovarian lesions (n = 3) in Javanese medaka at 21 days post-exposure to 3,4-DCA.

Treatment (µg/L)	Staging (Median)		Lesions (%)		
	^1^ Male	^2^ Female	Atretic OOCYTE	Oocyte Retraction	Karyoptic Clumping
Control	4	4	0.0	0.0	0.0
Solvent	4	4	0.0	0.0	0.0
10	4	4	0.0	0.0	1.8
25	4	3	2.1	5.2	7.3
50	3	4	4.9	1.5	3.0
125	4	4	1.3	0.0	5.7
250	3	2 *	11.2 *	5.2	4.7

* = significant difference at *p* < 0.05, % = percentage alteration in oocytes per section. ^1^ The guideline recommends the following gonadal staging scale for male: 0 = undeveloped, 1 = early spermatogenic, 2 = mid-spermatogenic, 3 = late spermatogenic, and 4 = spent. ^2^ The guideline recommends the following gonadal staging scale for female: 0 = undeveloped, 1 = early development, 2 = mid- development, 3 = late development, 4 = late development/hydrated, and 5 = post-ovulatory.

## Data Availability

The data presented in this study are available on request from the corresponding author.
